# The Patellar Arterial Supply via the Infrapatellar Fat Pad (of Hoffa): A Combined Anatomical and Angiographical Analysis

**DOI:** 10.1155/2012/713838

**Published:** 2012-06-06

**Authors:** Gregor Nemschak, Michael L. Pretterklieber

**Affiliations:** Center of Anatomy and Cell Biology, Department of Applied Anatomy, Medical University of Vienna, Waehringerstrasse 13, 1090 Vienna, Austria

## Abstract

Even though the vascular supply of the human patella has been object of numerous studies until now, none of them has described in detail the rich arterial supply provided via the infrapatellar fat pad (of Hoffa). Therefore, we aimed to complete the knowledge about this interesting and clinically relevant topic. Five human patellae taken from voluntary body donators were studied at the Department of Applied Anatomy of the Medical University of Vienna. One was dissected under the operation microscope, a second was made translucent by Sihlers-solution, and three underwent angiography using a 3D X-ray unit. The results revealed that the patella to a considerable amount is supplied by arteries coursing through the surrounding parts of the infrapatellar fat pad. The latter were found to branch off from the medial and lateral superior and inferior genicular arteries. Within the infrapatellar fat pad, these arteries formed a dense network of anastomoses which are all contributing to the viability of the patellar bone. Due to the rich arterial supply reaching the patella via the infrapatellar fat pad, it seems advisable to preserve the fat pad during surgery of the knee in order to reduce the risk of vascular impairment of the patella.

## 1. Introduction

The infrapatellar fat pad (of Hoffa) is located in the anterior compartment of the knee joint and is bordered by the inferior pole of the patella, the patellar retinacula, the patellar tendon, the anterior part of the tibia, the anterior horns of the menisci, and the femoral condyles. Further, it is attached to the intercondylar notch of the femur by the infrapatellar synovial plica also referred to as ligamentum mucosum [1]. Because of its high amount of nerve endings, the infrapatellar fat pad may become source of anterior knee pain. As reported by Bennell et al., the application of hypertonic saline into the fat pad led to pain experience in healthy volunteers [2]. In rare cases, tumour-like lesions of the infrapatellar fat pad such as osteochondroma, pigmented villonodular synovitis, synovial cysts [3], and vascular malformation [4] may lead to anterior knee pain. Furthermore, the infrapatellar fat pad may be affected by postarthroscopic alterations, postsurgery fibrosis, and shear injuries [5].

As reported by Bohnsack et al., the fat pad plays a role in stabilizing the knee joint in the extremes of motion, especially during flexion angles of less than 20° and greater than 100° [6]. Due to surgical demand, it is often removed or at least partially resected during surgery in order to improve visibility to the joint. Medial arthrotomy made too close to the patella, wide resection of the fat pad, lateral retinacular release, and cauterization of the prepatellar vessels are known to jeopardize vascular supply to the patella [7]. Hoffa's fat pad is traversed by two vertically running arteries, which branch off from the superior medial and lateral genicular arteries. Both vertical arteries anastomose with the correspondent inferior genicular arteries. Additionally, an anastomosis is found between the lateral vertical artery and the anterior tibial recurrent artery next to the lateral margin of the patellar ligament. Posterior to the patellar ligament, the two vertical arteries are interconnected by two to three horizontal arteries which are found between the inferior pole of the patella and the tibial tuberosity [8]. Due to their anatomical proximity, the infrapatellar fat pad and the patellar tendon have a shared blood supply; the latter is derived from the inferior genicular arteries and laterally also from the anterior tibial recurrent artery [9].

The human patella is mainly supplied by five to six arteries forming an anastomotic network on the anterior aspect of the bone. Especially the descending genicular artery, the medial and lateral superior genicular arteries, the medial and lateral inferior genicular arteries, and the anterior tibial recurrent artery contribute to this so-called Rete patellae. These arteries are also involved in the supply of the distal parts of the femur and the proximal aspects of the tibia [10]. The two superior genicular arteries run along the superior pole of the patella forming an anastomosis together with the descending genicular artery anterior to the insertion of the quadriceps tendon. The inferior genicular arteries give off three branches; one ascends along the lateral border of the patella, one converges towards the anterior aspect of the bone, and the third transverse running branch anastomoses with the contralateral artery deep to the patellar tendon. These vessels are also called transverse infrapatellar arteries. Inside the infrapatellar fat pad, the transverse infrapatellar arteries give rise to small vessels, so called polar vessels, which contribute to the vascular supply of the distal half of the bone [11].

Therefore, the patella is supplied by two arterial pathways. First, small vessels enter the anterior aspect of the bone through vascular foramina located on the anterior surface. Additional supply is provided by polar vessels originating from the transverse infrapatellar branches of the two inferior genicular arteries. Due to its dual blood supply, there is anatomical evidence that the distal half of the patella is less endangered by avascular necrosis; on the contrary, pathologically the upper half may be separated from the blood supply by transverse patellar fractures [11]. Both arterial pathways arise from the peripatellar anastomic network [12].

In addition to the findings just mentioned, according to Björkström and Goldie, arteries piercing the quadriceps tendon and the adjoining synovial tissue may reach the patellar base. Furthermore, they identified arteries supplying the medial, lateral, and superior borders of the patella. The authors emphasize the existence of deeply situated peripatellar arteries which give rise to the aforementioned arteries [13]. Considering the findings of Howard et al. who evaluated the vascular supply of canine patellae, the vascular anatomy seems to be comparable to the situation in humans. Thus, the canine patella is supplied by numerous arterioles entering the bone along the medial, lateral, and dorsal aspects, respectively [14].

Hence, a lot of information concerning the arterial supply of the patella has hitherto been published, the topographical relationship between the feeding arteries of the patella and the infrapatellar fat pad has not been presented in details. Thus, the aim of this study was to clarify to which extent arteries supplying the patella are to be found within Hoffa's fat pad.

## 2. Material and Methods

For the presentation of the vascular course within the infrapatellar fat pad of Hoffa, five isolated corpora adiposa were studied at the Department of Applied Anatomy of the Medical University of Vienna. These specimens were taken from two female and three male voluntary body donators which had died at a mean age of 75 years. All parts of this study have been approved by the local ethical board (registration number 919/2010). The first two knee joints were taken from anatomic specimens that had been used in the student dissection courses and, therefore, were perfusion fixed with a mixture of 1.6% formaldehyde solution and 5% phenol solution. One specimen taken from a 79-year-old male individual was dissected layer by layer by means of a surgical microscope (Zeiss OPMI 11; Carl Zeiss GmbH, Vienna); during this procedure, for better visibility, the vessels were injected with Wright's eosin methylene blue solution (Merck, Art 1383). The injection was carried out with Insulin Syringes (BD Micro-Fine, 1 mL of 0.33 mm (29) × 12.7 mm. BD Medical-Diabetes Care Becton Dickinson France SAS, Le Pont de Claix, France). Microanatomical preparation was carried out by microsurgical forceps and scalpel blade number 15 (Aesculap, Aesculap AG, et. Co. KG.).

The second specimen taken from an 85-year-old female body donator underwent a special designed preparation following the method originally described by Sihler as modified by Liu et al. [15, 16]. By this method, soft tissue was made translucent and thus a better representation of the vessels was achieved. First, the specimen was immerged in 10% nonneutralized paraformaldehyde for at least 1 month. Then, the fixed specimen was macerated. Maceration was started with washing in tap water for 30–60 minutes. Then, the specimen was digested for at least 3 weeks in 3% NaOH solution at refrigerator temperature. The macerating solution was changed daily until all parts of the specimen became translucent or transparent. In the following step, the specimen was decalcified. For this, Sihlers solution I was to be used (one part concentrated acetic acid, one part glycerol, and six parts of an aqueous solution containing 1% chloralhydrate). This step was to be continued until the cartilage was soft while the specimen is stored in the refrigerator. Finally, the tissue was stained in Sihlers solution II. This consists of one part concentrated EHRLICH hematoxylin, one part glycerol, and 6 parts of a 1% aqueous chloralhydrate.

Staining was carried out until the specimen became deep red. Furthermore, contrasting was achieved using Sihlers solution I again at room temperature. On a rocker table working at 200 rpm, the specimen was stored in the solution until it turned purple, then the solution was changed. Then, the specimen was washed in running tap water and then stored in a 0.05% lithium carbonate solution for one hour. Finally, transparency was reached by rewashing the specimen in running tap water for 30–60 minutes. Thereafter, the tissue block was stored in glycerol at ascending concentration of 40%, 60%, 80%, and at least in 100%. Thymol crystals were added to each series. Photographic documentation of both preparations was performed using a digital full-frame photo camera (Canon EOS 5D, Canon Inc., Tokyo, Japan) mounted on the camera tube of the operation microscope. For documentation, the translucent specimen was situated on a transparent slide screen (Kaiser slimlite, Kaiser Fototechnik GmbH & Co KG, Buchen, Germany) and illuminated from below. For angiographic examination, three fresh frozen anatomic specimens of human legs were used. They were taken from two male and one female individual which had died, on average, at the age of 71 years. In the region of the canalis vastoadductorius (Hunter's canal), the femoral artery was punctured with a Butterfly (Vacutainer System) and an iodine-containing contrast agent (Iomeron^®^) was injected. Subsequently, the three knee joints underwent 3D radiologic imaging (Siemens Arcadis Orbic 3D, Siemens Medical Solutions, Erlangen, Germany). This specially designed scanner obtained serial cross-sections in a volume of 12 × 12 × 12 centimeters by turning round the specimen by 190 degrees. According to the basic adjustment, parallel to each of the three planes 256 images were obtained as partly overlapping multiplanar reconstructions (MPR) with a slice thickness of 0.5 mm. Finally, the series of cross-sections were evaluated on the work station operating the scanner in order to trace the arteries marked by contrast media.

## 3. Results

**Figure 1:**
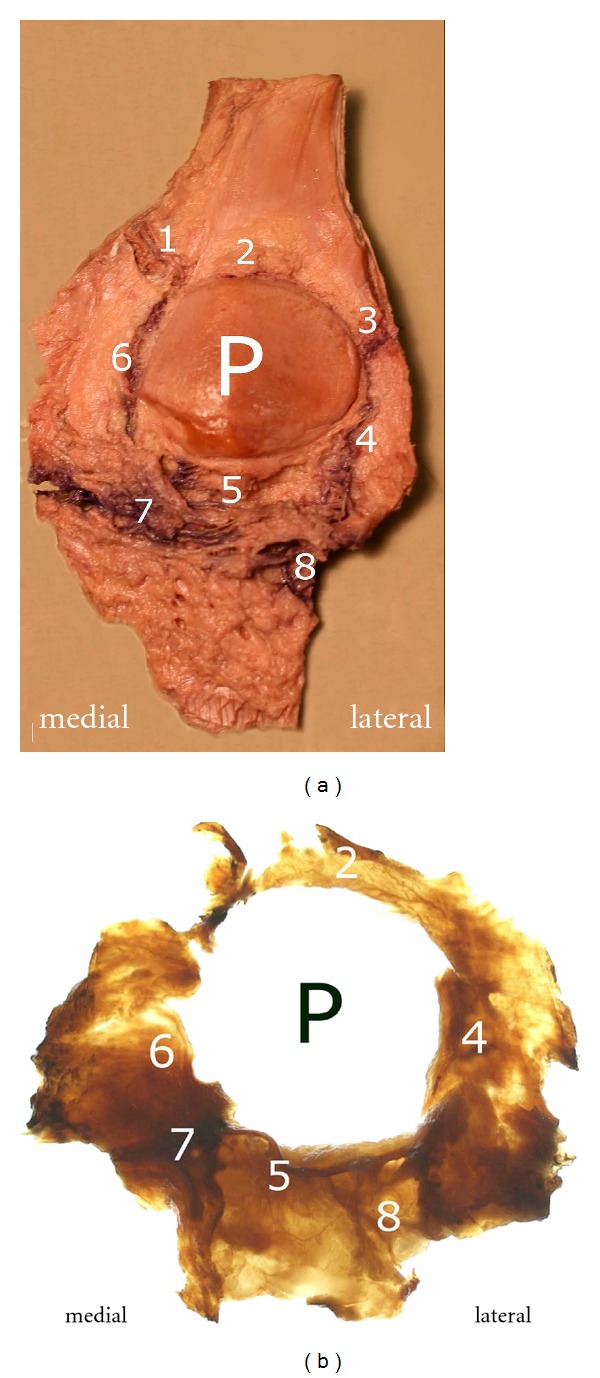
(a) and (b): Posterior view of the dissected fat pad (a) and the isolated translucent fat pad (b) as result of Sihler's procedure: (1) branches of the superior medial genicular artery, (2) anastomosis formed by the “basic branches” of the two superior genicular arteries, (3) branches of the superior lateral genicular artery, (4) descending branch arising from the superior lateral genicular artery, (5) deep vascular layer with transversely extending arteries, (6) descending branch from the superior medial genicular artery, (7) intermediate vascular layer, (8) branch of the anterior tibial recurrent artery and its entry into the vascular arcades of the infrapatellar fat pad.

**Figure 2:**
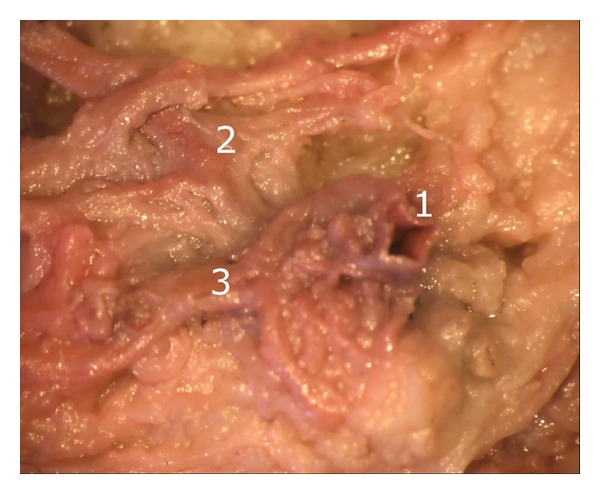
Termination of the middle genicular artery into the intermediate vascular layer: (1) terminal branch of the middle genicular artery, (2) and (3) vessels of the intermediate vascular layer.

**Figure 3:**
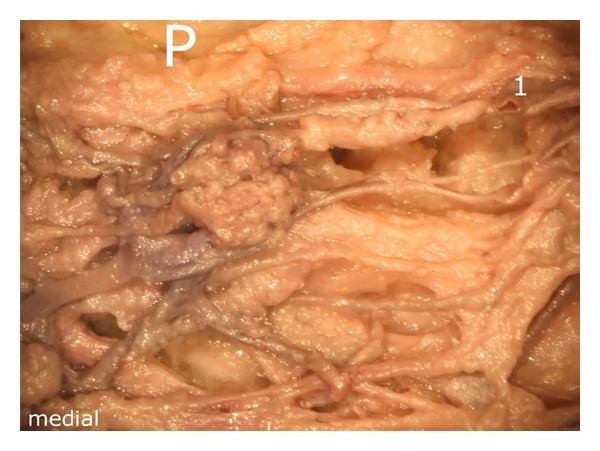
Representation of the intermediate vascular layer in the middle third of the fat pad, (1) afferent vessels from the depths of the knee joint (intercondylar fossa), P: patella.

After careful removal of the synovial membrane covering the inner aspect of the infrapatellar fat pad, a dense network of superficial vascular anastomoses appeared. These anastomoses were especially concentrated in the central parts of the fat pad including the distal portions adjacent to the patellar tendon. The superficial vascular plexus was formed by afferent vessels entering the fat pad from deep within the knee joint (intercondylar fossa); additional supply was received by branches of the inferior genicular arteries. The superficial vascular plexus only appeared to supply the fat pad itself and its synovial membrane, respectively, since no direct vascular connections to the bone could be detected. After removal of the superficial vascular plexus and careful microdissection of the remaining adipose tissue, a second layer of blood vessels appeared in the intermediate level of the fat pad (Figures 1 and 3). The vessels of the intermediate vascular layer themselves received their supply from the inferior genicular arteries, and laterally also from a branch of the anterior tibial recurrent artery. From within the intercondylar fossa, numerous vessels reached the dense anastomotic network in the central parts of the fat pad ((1) in Figure 2 and (c) in Figure 15(a)).

**Figure 4:**
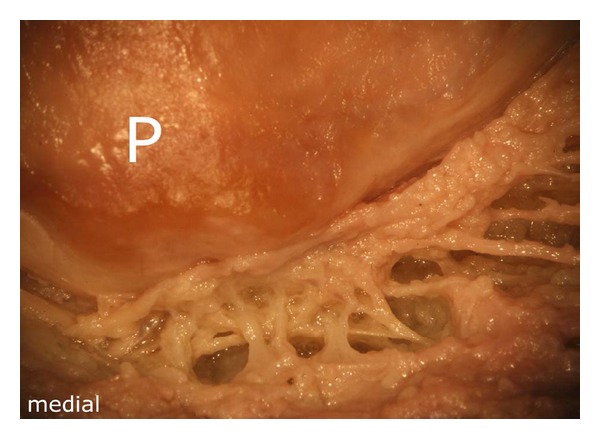
Distal pole of the patella. Note the numerous small vascular connections to the bone, which supply the lower half of the patella. The latter are derived from the intermediate vascular layer, P: patella.

The arteries running in the intermediate vascular layer supplied the distal patellar pole by numerous small vascular connections (Figure 4).

**Figure 5:**
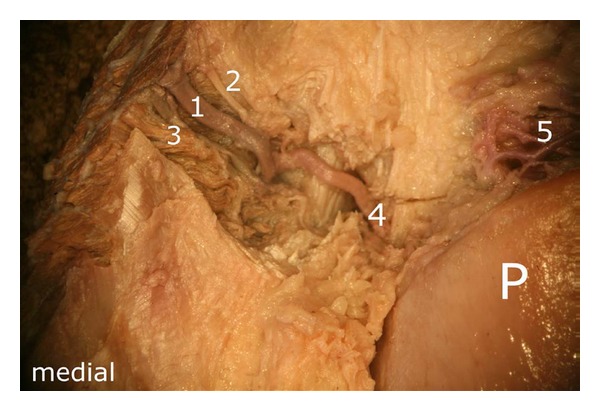
Branches of the superior medial genicular artery: (1) main vessel, widest in caliber, (2) and (3) accompanying vessels, (4) bifurcation of the main branch (1), (5) “basic branch” forming a dense anastomotic network next to the patellar base, P: patella.

The branches of the superior medial genicular artery ran towards the superior medial margin of the patella. Interestingly, as a constant finding in this part of the fat pad, a vessel with wider caliber was flanked by two smaller vessels in its course. After a short distance, the proximal vessel ((2) in Figure 5) joined the main branch ((1) in Figure 5). In the area of the medial insertion of the quadriceps tendon, the main vessel bifurcated ((4) in Figure 5).

The first branch ran directly to the anterior aspect of the quadriceps tendon and thus escaped from the preparation situs. The second branch originating from the bifurcation ran along the medial aspect of the patella, giving off numerous small branches, which supplied the medial border of the patellar bone. We found a small vessel arising from the descending branch which followed a tortuous course directed towards the superior pole of the patella ((5) in Figure 5). This vessel anastomosed with a similarly configured vessel arising from the superior lateral genicular artery. The distally located flanking vessel ((3) in Figure 5) joined the descending branch, after the superior polar vessel had been given off.

**Figure 6:**
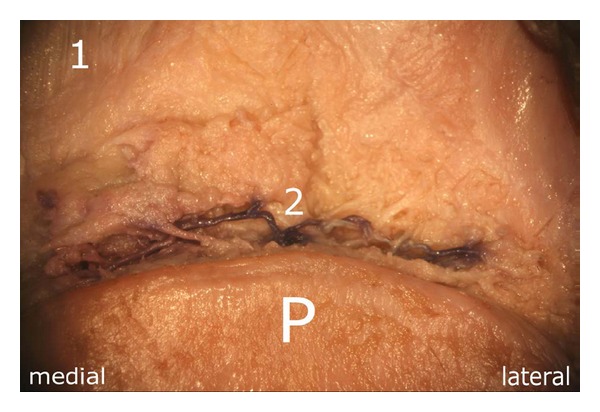
Anastomosis next to the superior pole of the patella. It is formed by the “basic branches” which arise from the two superior genicular arteries: (1) quadriceps tendon, (2) anastomosis, P: patella.

The previously mentioned formation of the anastomosis was found in the middle third of the superior pole of the patella, posterior to the quadriceps tendon ((2) in Figure 6). Before establishing this anastomosis, both arterial branches regularly delivered small vessels which contributed to the vascular supply of the superior pole of the bone.

**Figure 7:**
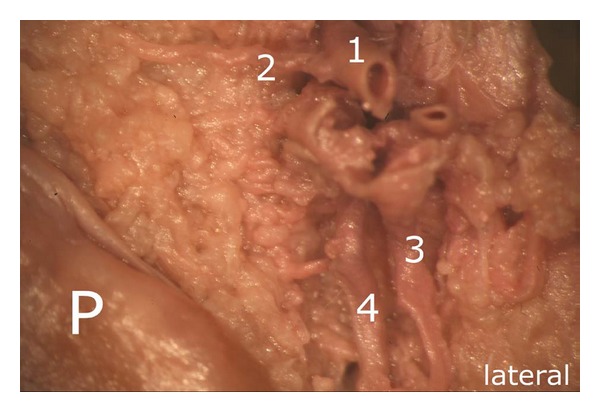
Branches of the superior lateral genicular artery: (1) runs towards the anterior aspects of the quadriceps tendon, (2) involved in the formation of the superior anastomosis, (3) flanking vessel which joins the descending branch (4), (4) descending branch forming an anastomosis with the ascending artery in the middle third of the lateral margin, P: patella.

The branches of the superior lateral genicular artery showed a configuration similar to that of the contralateral side. In the area of the superolateral border of the patella, we were able to identify three vessels which further contributed to the supply of the superior pole of the patella, including its lateral margin. The proximal of the three arteries ((1) in Figure 7) gave off a small branch ((2) in Figure 7) which ran towards the superior pole of the patella forming the superior anastomosis previously mentioned; thereafter, the proximal arterial branch escaped the preparation situs.

**Figure 8:**
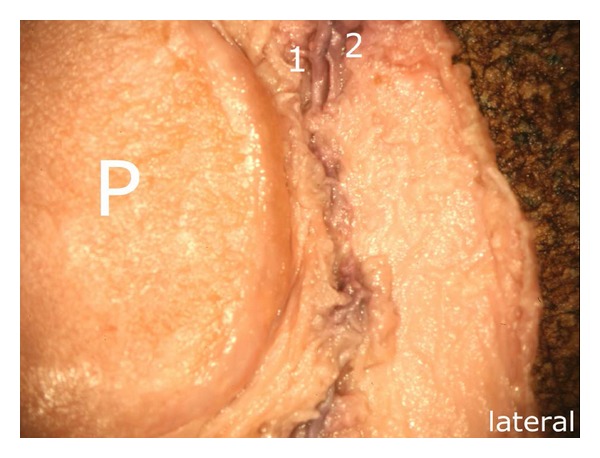
Course of the descending branch (corresponding to Figure 7): (1) descending branch arising from the superior lateral genicular artery (2) flanking vessel which joins the descending branch (1) P: patella.

**Figure 9:**
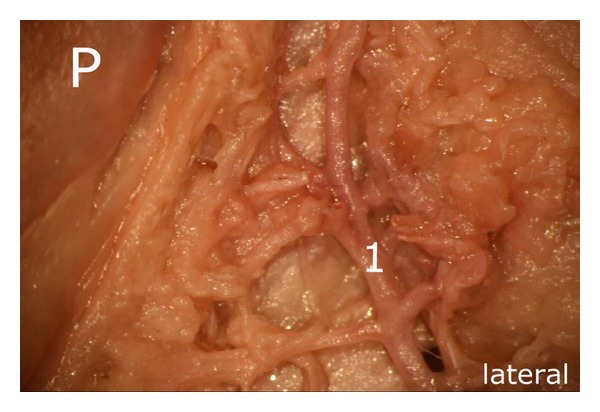
Note the small branches given off by the ascending artery (1) which are involved in the supply of the lateral aspect of the patella. This ascending artery departs from the lateral inferior genicular artery and anastomoses with the descending branch ((1) in Figure 8) of the superior lateral genicular artery, P: patella.

The descending branch ((4) in Figure 7) followed a strictly vertical course adjoining the lateral margin of the patella and delivered at regular intervals small vessels which supplied the lateral patellar margin. In the area of the middle third of the lateral margin, it formed an anastomosis with an ascending artery (Figure 8) which, in turn, arose from the inferior lateral genicular artery (Figure 9). Next to the superolateral patellar border, the descending branch was joined by another vessel ((2) in Figure 8).

Both the medial and lateral inferior genicular arteries supplied the patella through three different pathways. There was one branch running next to the border of the bone, another which converged to the anterior surface of the bone, and a third one running directly into the fat pad.

**Figure 10:**
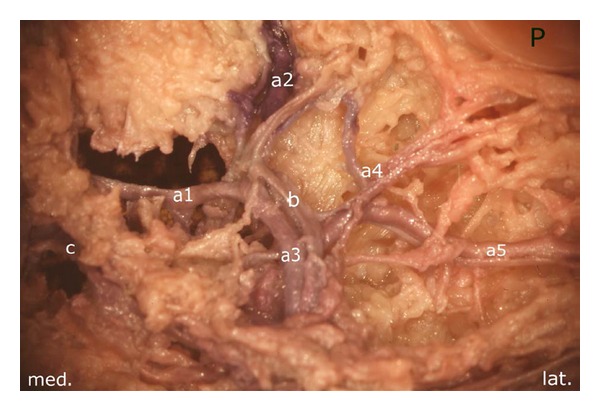
Medial aspect of the infrapatellar fat pad. Arteries arising from the inferior medial genicular artery. (a1) the main vessel gives off a proximal (a2), a distal (a3), a diagonally converging branch toward the patellar apex (a4) and a horizontal branch (a5). The latter is one of the strongest branches of the deep vascular level and forms an anastomosis with that of the lateral side. Except of (a5), all vessels are located in the intermediate vascular layer. P: patella.

**Figure 11:**
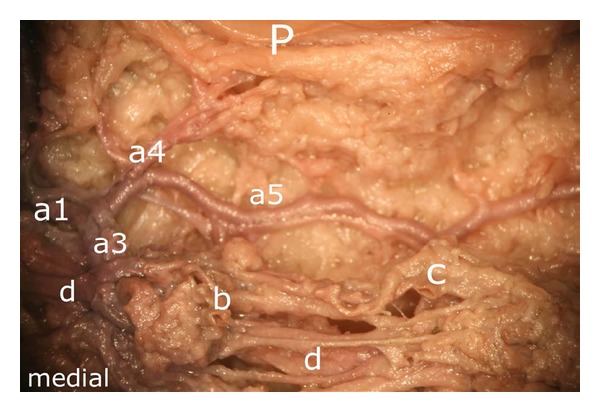
(In addition to Figure 10) Representation of the deep vascular layer (a5) and the intermediate one ((a1), (a3), (a4) and (d)). b and c mark terminal branches of the middle genicular artery, P: patella.

On a closer view, the transverse branch showed a very complex morphology, giving rise to numerous branches which were not limited to one level in their course. Figures 10 and 11 illustrate the three dimensionality of these vascular structures.

**Figure 12:**
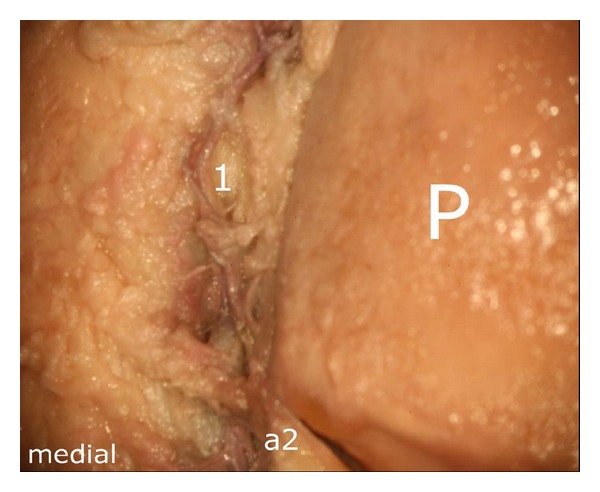
Anastomosis between the proximal branch of the inferior medial genicular artery (a2) and the descending branch of the superior medial genicular artery, (a2) proximal branch, (1) anastomosis, P: patella.

**Figure 13:**
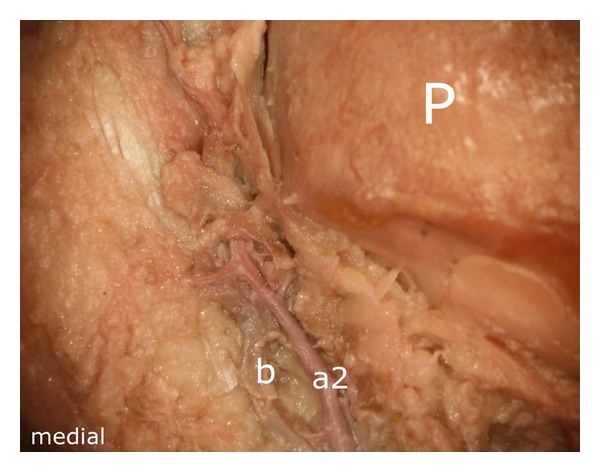
Ascending artery, derived from the inferior medial genicular artery ((a2) in Figure 10) (a2) ascending artery, (b) accomanying vessel which joins the main vessel (a2) at the distal medial margin of the patellar bone, P: patella.

In its further course, the proximal branch ((a2) in Figure 10) formed an anastomosis with the descending branch of the superior medial genicular artery at the level of the middle third of the medial patellar margin ((1) in Figure 12). The ascending artery was also accompanied by a smaller vessel ((b) in Figure 13) which joined the main ascending branch ((a2) in Figure 13).

**Figure 14:**
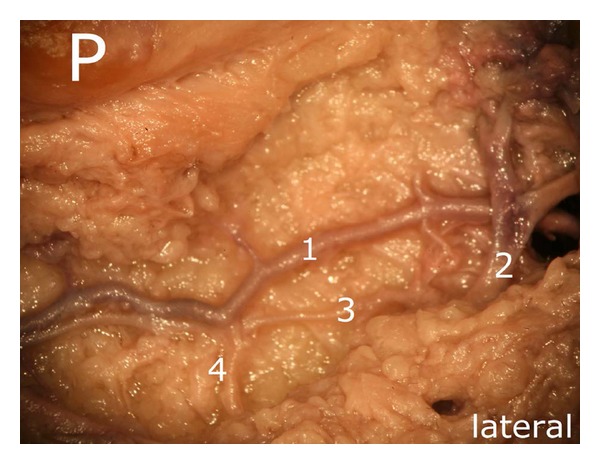
Deep vascular level, (1) vascular anastomosis of the deep level, out of the three it is the only one showing direct vascular connections to the bone; (2) one of the three anastomoses, (3) and (4) vascular connections between two anastomoses of the deep vascular layer, P: patella.

Considering the deep vascular level, we found an anastomosis in ultimate proximity of the patellar apex forming a vascular connection between the medial and the lateral inferior genicular artery ((1) in Figure 14).

**Figure 15:**
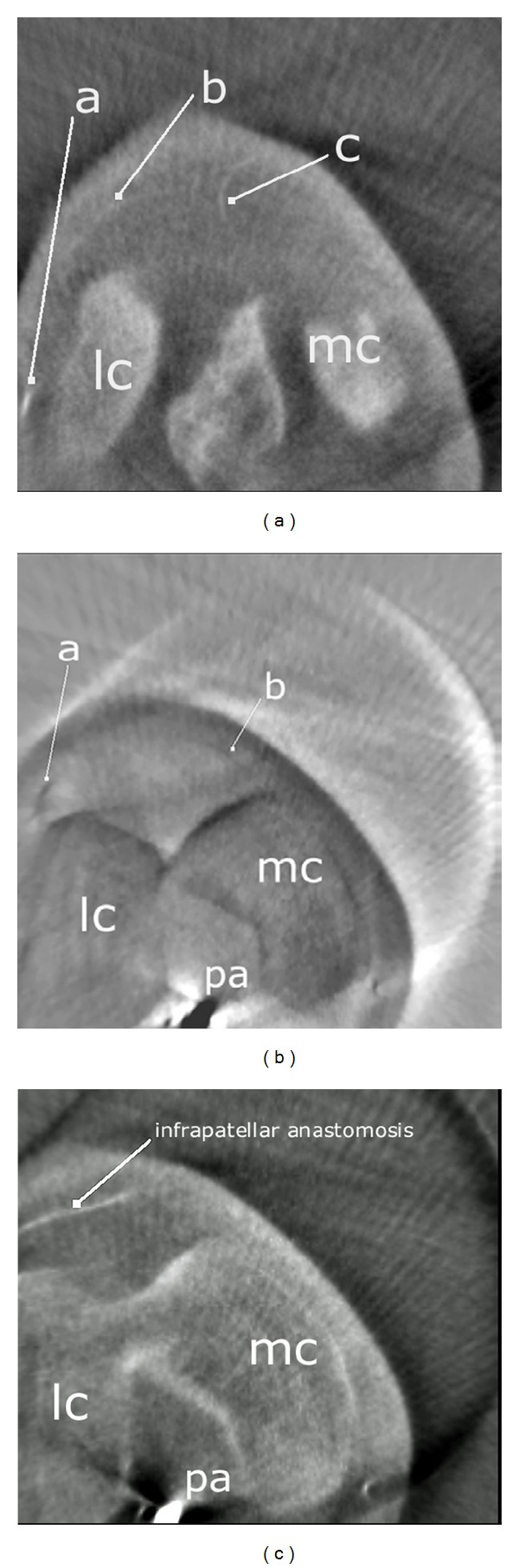
Axial MPR reconstructions of a right knee oriented in proximodistal sequence. (a) Intermediate vascular layer formed by a branch of the inferior lateral genicular artery and a terminal branch of the middle genicular artery. a: Inferior lateral genicular artery, b: arterial branch of the intermediate vascular layer, c: terminal branch of the middle genicular artery. (b) The inferior genicular arteries give rise to transversely running infrapatellar arteries, a: lateral transverse artery, b: medial transverse artery. (c) Transverse infrapatellar arteries anastomosing in proximity to the patellar apex. lc: lateral condyle of femur, mc: medial condyle of femur, pa: popliteal artery.

This anastomosis was also shown radiologically in Figure 15(c). Although two other anastomoses were found to be present, only the one next to the patellar apex showed direct vascular connections to the bone. Nevertheless, the three anastomoses were repeatedly linked to each other ((3) and (4) in Figure 14).

## 4. Discussion

The main findings of our study revealed that within the central mass of the infrapatellar fat pad, the vessels showed an arrangement of three layers, with the caliber of the vessels increasing from posterior to anterior, that is, towards the patellar tendon. Interconnections between these layers were established by numerous vascular channels. Together, they thus formed a functional unit. Except for the most superficial level, which was only found in the central portions of the infrapatellar fat pad or in the dorsodistal areas of the patellar tendon, both the intermediate and the deep vascular level provided vascular supply to the distal half of the patellar bone.

The vessels with the widest caliber were located in ultimate proximity to the posterior aspects of the patellar tendon and the patellar apex. The latter originated from the inferior genicular arteries and formed anastomoses within the fat pad. We, therefore, confirm the findings of Kohn et al. [8] who already described this vascular configuration previously, the same applies to the polar vessels described by Scapinelli [11]. However, our results showed that the transverse infrapatellar arteries established a complex branching pattern both medially and laterally. Especially noteworthy was the three dimensionality of the vascular architecture within the fat pad which had not been previously documented.

Our results revealed that, in the deep vascular layer, only one anastomosis supplied the inferior patellar pole directly by giving off small vessels. Nevertheless, it was found to be connected with the other anastomoses of the deep vascular layer ((3) and (4) in Figure 14).

As presented in Figure 10, we found a proximal, a diagonal (running towards the patellar apex), a horizontal (which anastomosed with the contralateral artery next to the patellar apex), and a distal branch (which, in its further course, turned into a vessel of the intermediate vascular level). On both sides of the patella, we found an ascending artery which anastomosed with the descending branch given off by the ipsilateral superior genicular artery in about the middle third of the lateral and medial margins of the patella posterior to the retinacula. Prior to this anastomosis, both the descending and the ascending arteries gave off small branches in their entire course at regular intervals, which supplied the lateral and medial edges of the patellar bone. Based on our results, we agree with the findings of Björkström and Goldie [13] that, besides the anterior vascular network [11] and the infrapatellar anastomoses, further arteries located posteriorly to the retinacula supply the medial and lateral borders of the patella. Interestingly, arteries originating within the quadriceps tendon had been described by other authors [13] but were not found to be present in our specimens. Instead of these vessels, in our specimens, an anastomosis was established by branches of the superior genicular arteries coursing next to the patellar base and posterior to the quadriceps tendon.

In the superomedial aspect of the patella, we found three parallel running branches originating from the superior medial genicular artery; one of them turned out to be the predominant vessel. After a short distance, the more proximally located branch joined the main branch. The main branch bifurcated next to the superomedial border of the patella. Whilst one branch ran to the anterior aspects of the quadriceps tendon and, therefore, was not available for further pursue, the other branch ran strictly vertical along the medial border of the patella. It soon gave off a small vessel, which followed a tortuous course directed towards the superior pole of the bone. The distally located flanking vessel joined the descending branch after the superior polar vessel had been given off. The branches of the lateral superior genicular artery showed a configuration which was very similar to that of the artery of the medial side. Both, the medial and the lateral superior polar vessels, formed an anastomosis in the superior aspect of the patella. Due to different techniques applied, this anastomosis together with its course had obviously been overlooked by other authors [10–13].

The results of our work indicate the existence of a dual supply of the entire patellar circumference, both anterior and posterior to the retinacula. The anastomoses within the infrapatellar fat pad established a vascular framing of the whole knee cap (Figures 1(a) and 1(b)).

In addition to the findings of Scapinelli [11], besides the anterior peripatellar ring, an additional arterial supply reached the patella via infrapatellar fat pad and was not limited to its central parts but also found within its superior parts and the alar folds.

Interestingly, the amount of anastomoses found in the specimen which was treated by Sihler's method was not as impressive as the results of the microsurgical dissection, although the vascular architecture behaved very similar to the the aforementioned specimen. According to Slater et al. [17], this may be due to the relatively high degree of retropatellar arthrosis this individual seemed to have suffered from.

Due to the small vessels under consideration as well as to a high amount of arteriosclerotic changes, angiographically we were only able to trace the main arteries contributing to the patellar supply.

Although numerous surgical interventions are known to jeopardize the vascular supply of the patella [7], obviously not every intervention inevitably leads to vascular impairment. Due to its characteristic arterial supply, the patella can thus assume a certain degree of tolerance.

However, if a medial parapatellar approach is combined with a lateral release, this may lead to an insufficient supply of the patella and subsequently increase the incidence of bone necrosis [18] and patellar fractures [19]. Preservation of the superior lateral genicular artery during lateral release as well as preservation of the infrapatellar fat pad is considered inter alia as reliable options in order to avoid patellar fracture resulting from total knee arthroplasty [20]. The superior lateral genicular artery may easily be detected in the subsynovial layer 1-2 cm distal to the inferior margin of the vastus lateralis muscle [21]. When arthroscopically performing a lateral release, Vialle et al. emphasized selective hemostasis of the superior lateral vascular pedicle and visualization of the inferior lateral vascular pedicle in order to minimize the risk of hemarthrosis which had been reported to occur in 10 to 18% of cases [22].

Nicholls et al. compared the reduction of blood flow to both the medial and lateral access but did not find any significant difference. From this, they concluded that both the medial and lateral arteries were involved equally in the vascular supply of the patella. Blood flow was found to be reduced to 53% following a medial approach and 27% after a lateral approach. The supply of the patellar tendon was not affected by both approaches. As the infrapatellar fat pad was preserved in all patients, due to its high amount of anastomoses, great importance is attributed to this structure on behalf of the vascular supply of the patellar tendon [23]. Significant shortening of the patellar tendon after resection of the infrapatellar fat pad was presented in two articles [24, 25]. As reported by Takatoku et al., postsurgical scar formation after resection of the infrapatellar fat pad led to abnormal shortening of the patellar tendon as well as deforming of the patella in growing rabbits. Moreover, preservation of the infrapatellar fat pad seems to prevent early degeneration of the articular surface of the patellofemoral joint [26]. Sanchis-Alfonso et al. observed the healing process of the patellar tendon after patellar tendon autograft and concluded that the infrapatellar fat pad as well as the paratenon played an important role in the healing process of the patellar tendon [27].

Based on a study in monkeys, Ogata et al. reported that performing a medial parapatellar approach which included a partial resection of the medial portion of the fat pad reduced the blood flow to the patella to 65% of the control animals. If the fat pad was completely removed, the blood flow decreased further to 49% of the control value. Total fat pad removal combined with a lateral release reduced blood flow to a dramatic low amount of 17% of the control value [28].

Another procedure which might endanger the patellar supply is the still controversially discussed need for patellar resurfacing. Proponents of resurfacing have justified this procedure with a lower incidence of revision surgery due to persistent anterior knee pain [29]. In addition, others have argued that forgoing patellar resurfacing has reduced the risk of patella fractures and component loosening [30].

Eversion of the patella is typically performed to optimize the operation field. However, as reported by Hasegawa et al., patellar eversion has led to a significant reduction of the patellar blood flow [31]. Concerning patellar denervation performed as a part of open knee surgery, in another study, no significant differences in outcome between denervated and nondenervated patellae could be detected during a 2-year followup period [32].

There are several reasons why we recommend preserving the infrapatellar fat pad especially during total knee arthroplasty: first we believe, taking our results into consideration, that the feeding arteries provided via Hoffa's fat pad play an important role in patella viability, as they may compensate the loss of other feeding arteries caused by invasive access routes. Second, shortening of the patellar tendon may arise with fat pad resection [24–26]. Third, fat pad resection has an influence on the biomechanics of the knee joint [33], as it seems to stabilize the knee joint, especially during extremes of knee motion (flexion angles of less than 20° and greater than 100°) [6].

However, if parts of the fat pad have to be resected, we emphasize sparing at least those parts of the fat pad which are located in ultimate proximity to the patellar borders. As far as the central mass of the fat pad is concerned, the fatty tissue next to the patellar tendon should be spared as the strongest anastomoses have been found to be present in this segment which, in turn, are also an integrative part of the arterial supply of the patellar tendon [34].

Weaknesses of our study include the small number of cases, so that no reliable conclusions can be drawn with respect to variations of the vascular anatomy within the infrapatellar fat pad (of Hoffa). Counting from another publication, the number of anastomoses varies depending on the degree of retropatellar arthrosis [17]. Furthermore, definite conclusions about the percentage of blood supply which reaches the patella via infrapatellar fat pad cannot be drawn. Further studies are needed in order to better estimate to which extent the anastomoses within Hoffa's fat pad are in a position to compensate the loss of other feeding arteries.

## 5. Conclusions

Until now, the arterial blood supply reaching the patella via the infrapatellar fat pad (of Hoffa) has not been subject of any detailed anatomical dissection, although radiographic presentations have been previously published [7, 11, 13]. Using microanatomical dissection techniques and a sophisticated morphological method to generate translucent specimens as well as multiplanar reconstructed angiograms, we were able to demonstrate the course of anastomoses in the peripatellar portions of the fat pad, especially in its superior aspect, which have obviously been overlooked in previous articles.

Advancing minimally invasive access routes are certainly demanded in nowadays surgery and mainly claim to reduce complications of any kind. Since various surgical procedures might endanger patellar vascular supply, each step should be weighed critically in order to achieve best possible outcome for the patients.
